# Exploring neuropsychiatric manifestations of vitamin B complex deficiencies

**DOI:** 10.3389/fpsyt.2025.1569826

**Published:** 2025-08-19

**Authors:** Anamaria Han, Leandro Almeida, Nikhilesh Anand, Ihsan M. Salloum, Salim Kanaan, Bharathi S. Gadad, João Paulo Lima Daher

**Affiliations:** ^1^ Faculty of Medicine, Universidade Federal Fluminense, Niterói, Brazil; ^2^ Department of Medical Education, University of Texas Rio Grande Valley (UTRGV) School of Medicine, Edinburg, TX, United States; ^3^ Institute for Neurosciences, University of Texas Rio Grande Valley (UTRGV) School of Medicine, Harlingen, TX, United States; ^4^ Department of Pathology, Faculty of Medicine, Universidade Federal Fluminense, Niterói, Brazil

**Keywords:** thiamine, riboflavin, niacin, pyridoxine, folate, cobalamin, complex B vitamins, neuropsychiatric manifestations

## Abstract

B complex vitamins, a group of eight water-soluble vitamins, play interconnected roles in maintaining nervous system health. Thiamine (B1), riboflavin (B2), and niacin (B3) are essential as co-enzymes in numerous metabolic reactions related to energy production. Thiamine is involved in the Krebs cycle, riboflavin in the electron transport chain, and niacin plays a key role in both glycolysis and the Krebs cycle. These metabolic processes are vital for sustaining the integrity of the nervous system, as the energy produced is critical for the functioning of nerve cells. Deficiencies in these vitamins can lead to significant neurological and psychiatric conditions, including Wernicke Korsakoff syndrome, Parkinson’s disease, and various mental illnesses. Additionally, pyridoxine (B6), folate (B9), and cobalamin (B12) are indispensable coenzymes for the methylation of homocysteine to methionine, a process critical to nervous system function. Elevated homocysteine levels, resulting from deficiencies of these vitamins, are associated with higher risks of depression and dementia. Thus, imbalances in these vitamins can disrupt key biochemical pathways, leading to neuropsychiatric disorders. The literature reviewed underscores the importance of daily intake of B complex vitamins to maintain normal serum levels and optimal neuronal function. This review aims to elucidate the neuropsychiatric manifestations associated with deficiencies in these vitamins.

## 1 Introduction

**Figure 1:**
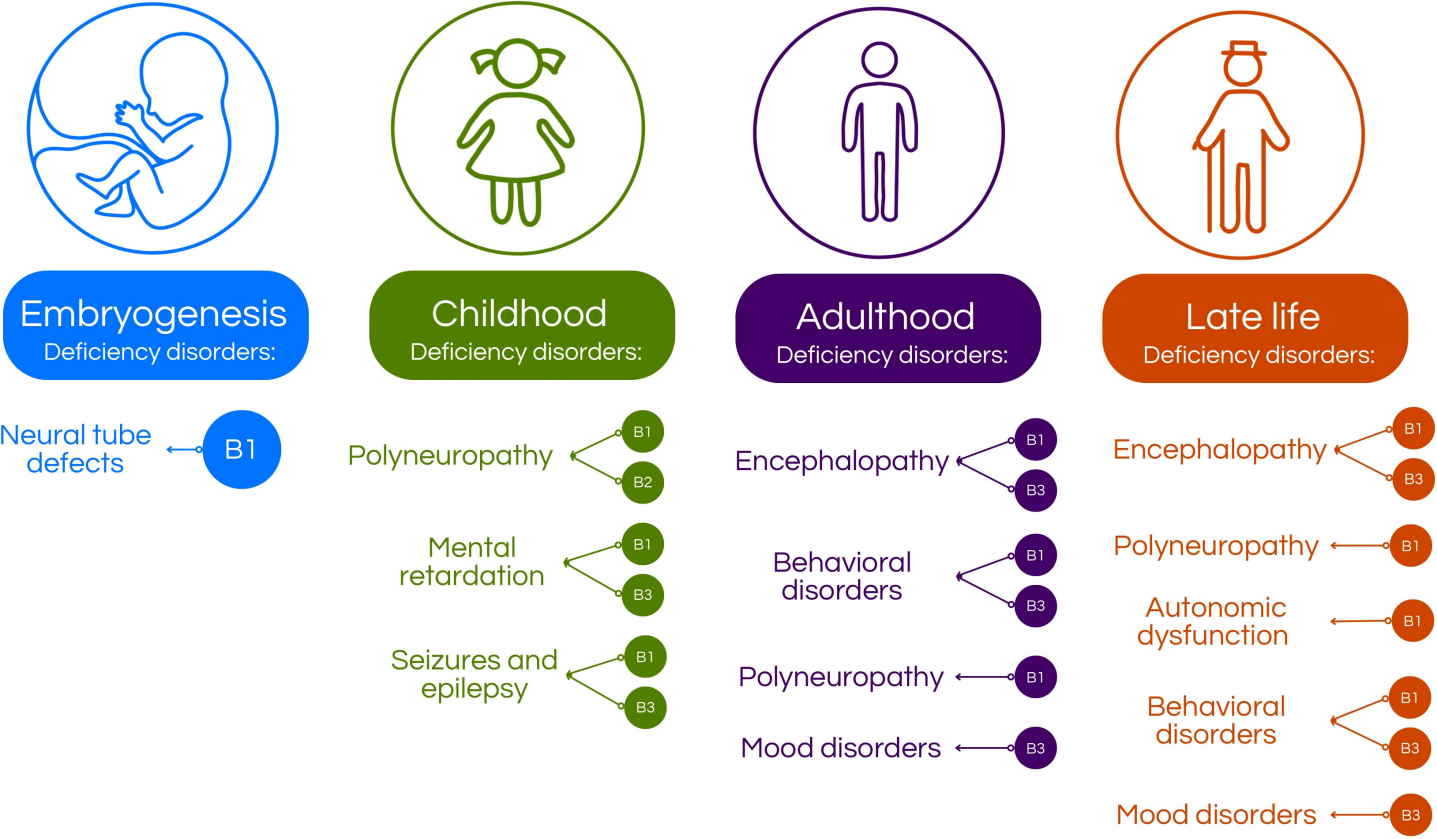
Age-related neuropsychiatric manifestations associated with B-vitamins deficiency. In the embryogenesis, B1 deficiency may cause defects in neural tube formation (5). In children, deficiency of B-vitamins may cause intellectual disability, polyneuropathy or seizures and epilepsy, depending on which vitamin is deficient (5–7). In adults, the deficiency may present in form of behavioral disorders, mood disorders, encephalopathy and polyneuropathy (5–8). Then, 5 of 29 on the elderly, B-vitamins deficiency may manifest as mood disorders, behavioral disorders, autonomic dysfunction, polyneuropathy and encephalopathy (5, 8–12).

**Figure 2:**
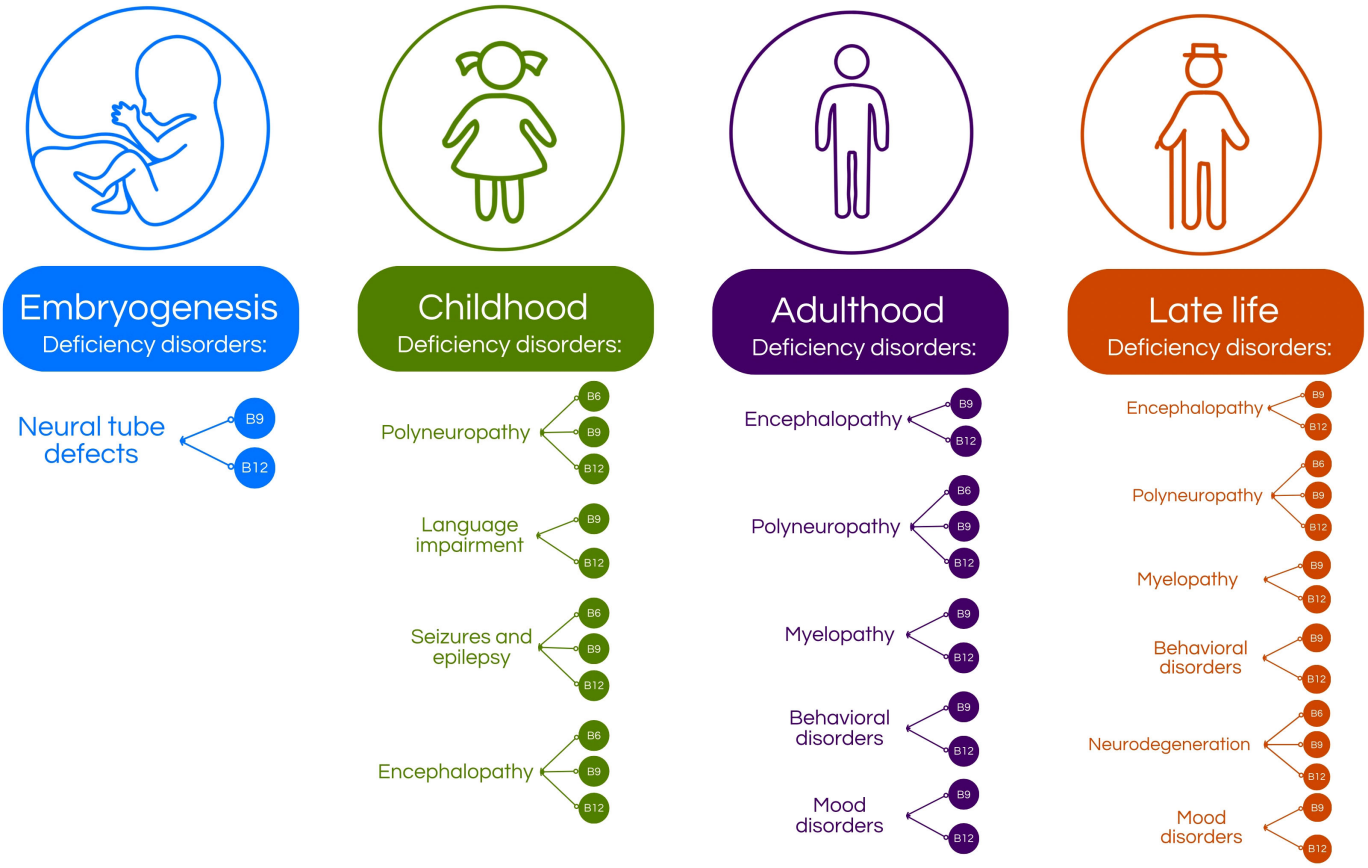
Age-related distribution of vitamin B deficiencies and their associated nervous system disorders. During embryogenesis, deficiencies in vitamins B9 and B12 are linked to the development of neural tube defects (4, 5, 13–16). In childhood, deficiencies in vitamins B6, B9, and B12 can lead to neurological conditions such as polyneuropathy, seizures, epilepsy, and encephalopathy (4, 5, 8, 10, 17, 18). Additionally, vitamin B9 and B12 deficiencies may contribute to language development impairments (4, 5, 8, 10, 17, 18). In adulthood, vitamin B9 and B12 deficiencies are associated with encephalopathy, myelopathy, and various behavioral and mood disorders, while deficiencies in B6, B9 and B12 can also cause polyneuropathy (4, 5, 19–24).

Vitamins of the B complex comprise a group of eight water-soluble vitamins that play a crucial role in maintaining optimal neurological function, such as energy production, DNA/RNA synthesis/repair, genomic and non-genomic methylation, and synthesis of neurochemicals (1–4). Consequently, deficiencies of these essential vitamins can lead to a spectrum of neuropsychiatric manifestations (1–4). Major neurological disorders associated with deficiencies of one or more B vitamins are often part of a broader set of organ complications since they are not the leading cause, but a manifestation of more significant underlying issues (5). These disorders encompass a broad spectrum of pathological conditions, show extreme phenotypic variation, and have roughly age-related distribution (**Figures 1**, **2**) (5). This review aims to shed light on the intricate relationship between vitamin B deficiencies and their impact on neuropsychiatric health.

Thiamine (B1) deficiency is often associated with beriberi, a syndrome mainly characterized by polyneuritis and/or cardiovascular symptoms (6–8). Its suggested daily intake varies from 1.1 to 1.2 mg/day (1, 7, 8). Beriberi can result in cognitive impairment, memory loss, and ultimately Wernicke-Korsakoff syndrome (6–8). The latter, characterized by confusion, ataxia, and oculomotor abnormalities, highlights the importance of thiamine in preventing severe neurological complications (5, 6). Riboflavin (B2) deficiency has been linked, in some ongoing research, to mood disorders and cognitive decline, as well as Parkinson’s Disease and depression (1, 9). Vitamin B2 daily intake is recommended to be 1.1 to 1.3 mg/day (5). Its role in energy metabolism underscores its significance for proper brain function (9, 10). Pellagra, a niacin (B3) deficiency disease, presents with neuropsychiatric symptoms such as dementia, anxiety, and depression (5, 11, 12). Niacin’s involvement in neurotransmitter synthesis, such as dopamine, emphasizes its impact on mental health such as major depressive disorder, anxiety and its deficiency can lead to neurodegenerative disorders (5). The suggested daily intake of niacin is 15 to 20 mg/day (5, 11, 13–15). Pyridoxine (B6) deficiency may lead to irritability, depression, and cognitive impairment, as well as epilepsy and cerebrovascular diseases (4, 6, 11, 16). The role of B6 in serotonin and dopamine synthesis underscores its importance in maintaining mood stability and cognitive function (17, 18). The recommended intake of pyridoxine is 1.3 mg/day (1). Folate (B9) deficiency has been associated with an increased risk of depression and cognitive decline, as well as with spinal cord and peripheral nerve diseases, organic brain syndrome, autism spectrum disorders and Alzheimer’s disease (4, 6, 11, 16–20). Adequate folate levels are essential for neurotransmitter synthesis, such as dopamine and serotonin, and regulation of homocysteine levels, the latter linked to neurodegenerative disorders (4, 20–24). Folate intake is suggested to be 400 µg/day (1, 22). Vitamin B12 deficiency is well known for causing neurological damage, often presenting as peripheral neuropathy and cognitive decline, as well as for the development of cerebrovascular, Parkinson’s, and Alzheimer’s diseases (20, 25–27). Moreover, the association between B12 deficiency and psychiatric disorders, mainly depression and psychosis, highlights the crucial role of this vitamin in maintaining mental health (20, 26, 27). Its recommended intake is 2.4 µg/day (1).

In conclusion, B vitamins are essential for both catabolic and anabolic metabolism functioning as coenzymes in numerous processes vital to cellular physiology, including brain and nervous system activities (5). The intricate relationship between the vitamin B complex and neuropsychiatric health underscores the necessity of maintaining adequate levels of these nutrients. Since they are not synthesized by the human body, except for niacin, they must be obtained through frequent dietary intake, since they are water-soluble (5). An unbalanced supply of a single B vitamin can obscure deficiencies in other B vitamins, potentially leading to persistent cellular energy failure and subsequent neurological damage (5). Early recognition and treatment of these deficiencies can help prevent and manage various neuropsychiatric conditions, thereby enhancing overall mental health (6). In patients with multiple B vitamin deficiencies, treatment failure may result from inadequate dosing or from a lack of success in identifying concurrent deficiencies in other B vitamins (1). Since B vitamins act synergistically in cellular physiology, understanding the specific roles of each vitamin can guide effective treatment strategies (5). This article aims to elucidate the biochemical correlations between B complex deficiencies and neuropsychiatric manifestations (**Table 1**). Further research is necessary to enhance our understanding of these relationships and to investigate novel therapeutic interventions for individuals with vitamin B deficiencies and associated neuropsychiatric symptoms.

**Table 1:** Recommended daily intake for each B complex vitamin and the associated neurological and psychiatric manifestations resulting from their deficiencies.

Vitamins	Recommended daily intake	Neurological manifestations in deficiency	Psychiatric manifestations in deficiency
Thiamine (B1)	1.1 – 1.2 mg/day (1, 7, 8)	Confusion, psychomotor retardation, impaired memory, and cognitive function, ataxia, burning feet, Wernicke’s encephalopathy, and Korsakoff’s syndrome (5–8, 36, 40).	Short-term memory loss and hallucinations (Korsakoff Psychosis) (40, 42)
Riboflavin (B2)	1.1 – 1.3 mg/day (5)	Neonatal mitochondrial encephalopathy, sensorineural hearing loss, axonal neuropathy, motor neuron disease, migraine headaches and Parkinson’s Disease (9, 47).	Mental illness and depression (48, 49).
Niacin (B3)	15–20 mg/day (5, 11, 14)	Neurodegenerative disorders, Pellagra with dementia, headache, myoclonus and ataxia (5, 11-13, 15, 53, 55).	Memory loss, depression, disorientation, and fatigue (5, 11, 12, 54)
Pyridoxine (B6)	1.3 mg/day (1)	Epilepsy, seizures, cerebrovascular diseases, paresthesias, painful dysesthesias, and Carpal Tunnel syndrome (4, 6, 11, 16–18, 58, 59)	Depression, cognitive impairment, irritability (5, 11, 17, 18)
Folate (B9)	400 µg/day (1, 22)	Spinal cord and peripheral nerve disease, dementia, organic brain syndrome, pyramidal tract damage, neural tube and brain development defects in the fetus, psychogeriatric disease, autism spectrum disorders, and Alzheimer’s disease (4, 19–21, 24, 30, 68, 69)	Depression, fatigue, apathy, insomnia, impaired concentration, impaired spatial copying skills, abstraction performance and non-verbal abstract thinking, irritability and decrease in intellectual function (4, 19, 30)
Cobalamin (B12)	2.4 µg/day (1)	Cerebrovascular disease, myelopathy, peripheral and autonomic neuropathy, progressive axonal demyelination, impaired peripheral and sensory nerve function, areflexia, loss of proprioception and vibration sensitivity, subacute combined spinal cord degeneration, Parkinson’s disease, and Alzheimer’s disease (25, 27, 64, 70)	Depression, dementia, episodes of psychosis, delirium, poor memory performance and cognitive impairment (20, 25–27, 64, 70)

## 2 Vitamin B1

Vitamin B1, or thiamine, is an essential nutrient obtained through dietary sources such as whole-grain products, brown rice, meat, vegetables, and fruits (1, 8). The recommended daily intake for thiamine in adults is 1.2 mg/day for males and 1.1 mg/day for females (1, 8, 28). A deficiency in vitamin B1 can develop rapidly, with inadequate nutritional intake over just two to three weeks, potentially leading to clinical symptoms of deficiency (1, 5, 8).

Thiamine is integral to the biochemistry of the nervous system, serving primarily as a coenzyme in key enzymatic reactions associated with energy metabolism, particularly the conversion of carbohydrates into usable energy (5, 7, 29). Furthermore, thiamine is critical for maintaining the integrity and proper function of nerve cells (1, 6, 30, 31). It plays a vital role in the maintenance of nerve membrane function, as well as in the synthesis of myelin and numerous neurotransmitters, including acetylcholine, serotonin, and various amino acids (6, 8, 30, 31). These functions collectively contribute to the structural and functional integrity of neurons and neuroglia (3, 6, 32).

**Figure 3:**
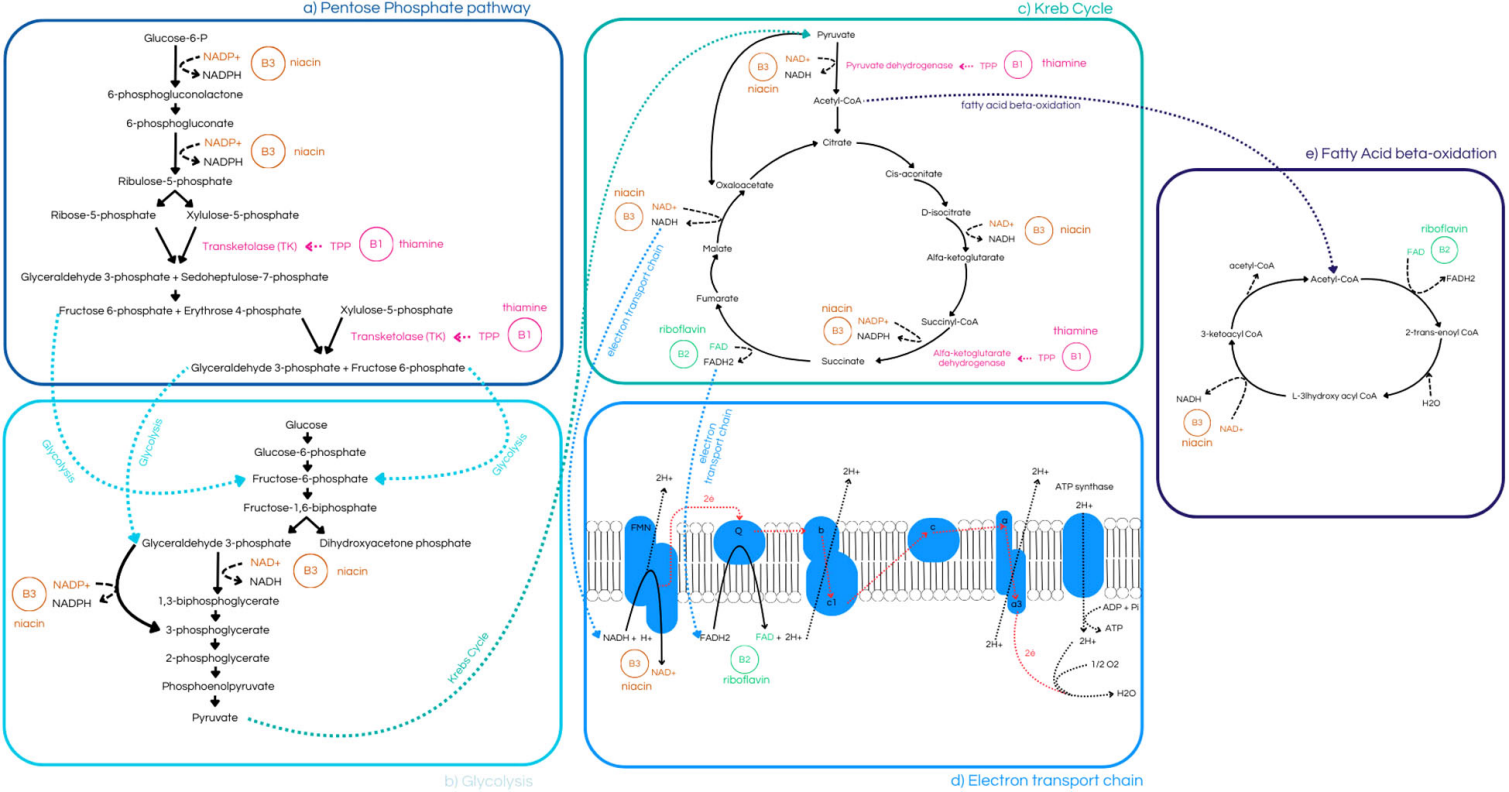
**(a)** Pentose Phosphate Pathway, highlighting the role of niacin (B3) in the synthesis of 6-phosphogluconolactone and ribulose-5-phosphate, as well as thiamine’s (B1) involvement in the production of 9 of 29 transketolase (TK) (8, 30, 31, 34, 35); **(b)** Glycolysis pathway, demonstrating niacin’s (B3) role in the conversion of 1,3-bisphosphoglycerate to 3-phosphoglycerate, and its links to both the Pentose Phosphate Pathway and the Krebs Cycle (34, 35); **(c)** Krebs Cycle, illustrating its connection to glycolysis, Electron Transport Chain, and Fatty Acid Beta-Oxidation pathway. It highlights thiamine’s (B1) role in catalyzing enzymes such as Pyruvate Dehydrogenase and α-Ketoglutarate Dehydrogenase, **(e)** riboflavin’s (B2) contribution to fumarate production, and niacin’s (B3) importance in the synthesis of α-Ketoglutarate and succinate (8, 10, 30, 31, 33–35); **(d)** Electron Transport Chain, emphasizing niacin’s (B3) role in redox reactions and riboflavin’s (B2) function as an electron-shuttling cofactor (3, 33–36).

In the brain, thiamine is phosphorylated at the cellular level to form its active derivative, thiamine pyrophosphate (TPP) (6, 11, 33). TPP functions as a cofactor for several critical enzymes across three metabolic pathways: transketolase (TK) in the pentose phosphate pathway, pyruvate dehydrogenase (PDH) in glycolysis, and alpha-ketoglutarate dehydrogenase (AKD) in the Krebs cycle (6, 33, 34). These pathways facilitate the production of energy in the form of adenosine triphosphate (ATP) and nicotinamide adenine dinucleotide phosphate (NADPH), both of which are essential for numerous cellular reactions (34, 35). Thus, thiamine, combined with other vitamins of the B complex such as B2 and B3, is vital for the proper functioning of various enzymes involved in key biochemical processes, primarily those related to energy production (6, 34, 36) (**Figure 3**).

The pentose phosphate pathway, through the action of transketolase (TK), converts ribose-5-phosphate to glyceraldehyde-3-phosphate (6, 34, 37). The substrates generated in this pathway are essential for synthesizing numerous molecules, including nucleic acids, steroids, and neurotransmitters (34, 37). Additionally, TK links this pathway to glycolysis, which enables the cell to adapt to its metabolic needs (37, 38). Pyruvate dehydrogenase (PDH) and alpha-ketoglutarate dehydrogenase (AKD) play pivotal roles in glycolysis and the Krebs cycle, respectively, since AKD, in the Krebs cycle, helps maintain neurotransmitter levels and supports protein synthesis (37, 39, 40). PDH catalyzes the formation of acetyl coenzyme A, a crucial step in energy production, and, through acetyl-CoA, serves as a precursor for acetylcholine (ACh), which is vital for nerve signal transmission and muscle function since ACh acts on the communication between neurons and in the neuromuscular junction, as well as for myelin production in nerve cells (36, 39, 40). Thiamine deficiency adversely affects the activity levels of these enzymes, with TK, through thiamine-dependent erythrocyte transketolase (ETK) activity, common biomarkers of thiamine status, since it is particularly sensitive to thiamine levels and AKD often exhibiting the earliest detectable changes (6, 41).

Given its numerous functions, low levels of thiamine can lead to reduced energy production due to impaired mitochondrial function, which damages neurons, and cells that have high energy demands, making them particularly vulnerable to potential damage or cell death (3, 6, 7, 11). Thiamine deficiency can affect both the central nervous system (CNS) and peripheral nervous system (PNS), manifesting in various symptoms (6, 7, 40). General symptoms of thiamine deficiency include confusion, psychomotor retardation, impaired memory and cognitive function, and ataxia (6, 7, 36, 40). Polyneuropathy typically presents gradually, beginning with paresthesia known as “burning feet,” followed by gait disturbances and variable muscle weakness in the lower limbs (5, 7). In children, dysphonia may also occur (5, 7).

In the CNS, thiamine deficiency can lead to Wernicke’s encephalopathy, characterized by nystagmus, ophthalmoplegia, mental status changes, and unsteady stance and gait, due to apoptotic cell death in sensitive brain areas, mainly areas with high metabolic requirement, causing symmetrically distributed lesions among structures such as the third and fourth ventricles, besides dorsomedial thalamus (5, 6, 42). Despite this, 75-80% of cases are not diagnosed, since the classical triad of ataxia, eye signs, and confusion is found in only about 20% of patients. This underdiagnosis can lead, besides death in 20% when it is not appropriately treated, to Korsakoff’s syndrome, a severe dysfunction often following untreated Wernicke’s encephalopathy, that does not remit even with thiamine treatment and is associated with chronic alcohol abuse and chronic thiamine deficiency (5, 6, 11, 28). Risk factors for Korsakoff’s syndrome include repetitive thiamine deficiency episodes and genetic predispositions, such as mutations in the transketolase enzyme that reduce its affinity for TPP (5). It is characterized by short-term memory loss and hallucinations, known as Korsakoff Psychosis, as a consequence of permanent brain damage (40, 42).

In the PNS, thiamine deficiency can result in polyneuritis and paralysis, as seen in dry beriberi, which affects the sensory system, causing pain, altered temperature sensitivity, numbness, reduced tendon reflexes, and leg atrophy (6, 11, 36). Wet beriberi, which refers to infantile thiamine deficiency, is characterized by cardiomyopathy, heart failure, warm extremities, and pulmonary edema, with early symptoms including fatigue, nausea, and mental suppression (6, 7, 11).

Pan et al, in a *in vivo* study performed with transgenic mice, have shown that chronic treatment with benfotiamine, a thiamine derivate with better bioavailability, enhanced the spatial memory and reduced both amyloid plaque numbers and phosphorylated tau levels, suggesting that this thiamine derivative may be useful in clinical Alzheimer’s disease treatment, although others thiamine derivatives do not showed the same results (**Table 2**). Pan et al. has also shown, in a clinical study, that patients with mild to moderate dementia manifested improved cognitive ability. The research was conducted with five subjects who received oral benfotiamine for 18 months.

**Table 2:** Recommended daily intake for each B complex vitamin and the associated neurological and psychiatric manifestations resulting from their deficiencies.

Authors	Supplemented vitamins	Type of study	Method	Findings
Pan, et al. (84)	Thiamine (B1)	*In vivo*	APP/PS1 double transgenic mice were used and assembled in groups that received different doses of thiamine derivatives.	The study found that in 8 weeks, benfotiamine (a thiamine derivative with better biodisponibility) enhanced spatial memory and reduced amyloid plaque numbers and phosphorylated tau levels in mice brains, which suggests that it can be beneficial for clinical Alzheimer’s disease treatment.
Pan, et al. (85)	Thiamine (B1)	Clinical study	Five patients with mild to moderate Alzheimer’s disease were treated with oral benfotiamine for 18 months.	The results showed that patients with mild to moderate dementia manifested a long-term improvement in cognitive ability using benfotiamine, although it showed an exacerbation of brain amyloid accumulation, showing that Alzheimer’s disease may be halted or improved, and the improvement of cognitive function is independent of brain amyloid accumulation.
Hoane, et al. (86)	Riboflavin (B2)	*In vivo*	41 male Sprague-Dawley rats received contusion injuries or sham procedures. Following injuries, 7 rats received riboflavin and 8 rats received saline in two different periods of time.	The study showed that riboflavin has therapeutic potential to Traumatic Brain Injury, since it has shown that vitamin B2 reduced the behavioral impairments and improved the acquisition of both reference and working memory tests compared to saline-treated rats, while also reducing the size of the lesion.
Wang, et al. (87)	Niacin (B3)	*In vitro* and *in vivo*	*In vivo* study used Alzheimer’s disease (AD) model rats and *in vitro* study used organotypic hippocampal slice cultures to investigate the effects of nicotinamide mononucleotide on Aβ1–42 oligomer-induced neurotoxicity.	The study found that nicotinamide mononucleotide (NMN) ameliorated learning and memory on AD model rats. It reduced cell death on organotypic hippocampal slice cultures, as well as restored levels of NAD+ and ATP.
Giri, et al. (88)	Niacin (B3)	*In vitro* and clinical study	*In vitro* used macrophage RAW64.7 cells and the clinical study used 24 patients with Parkison’s disease compared to the same number of age-matched control group of subjects.	The study found that niacin reduced the translocation of phosphorylated nuclear kappa B in the nucleus of RAW264.7 cells, decreasing pro-inflammatory cytokines after the niacin’s receptor GPR109A knockdown. The niacin use reduced the GPR109A up-regulation in Parkinson’s disease patients and, thus, may reduce inflammation.
Yang and Wang (78)	Pyridoxin (B6)	*In vivo*	Used isolated nerve terminals purified from rat cerebral cortex to research the influence of pyridoxine on glutamate release.	The results demonstrated that pyridoxine inhibits glutamate release from cerebrocortical synaptosomes by suppressing voltage-dependent Ca2+ entry, which supports the idea that vitamin B6 can function as a potential source for brain therapeutics against excitotoxicity.
Weng, et al. (79)	Folate (B9)	*In vitro* and *in vivo*	The *in vivo* studies used folate- deficient mouse model to	The research found that folate- supplemented diets during pregnancy
			research the role of folate on oligodendrocyte development, giving normal folate- supplemented diets to some of the mice and low folate- supplemented diets to others. The *in vitro* studies used primary oligodendrocyte precursor cells (OPCs) isolated from neonatal rat brains and different concentrations of folate.	and lactation accelerates the maturation of oligodendrocyte by activating dihydrofolate reductase (DHFR). In addition, oligodendrocyte death and differentiation defects caused by pharmacological DHFR inhibition can be rescued by folate supplementation. Finally, folate/DHFR signaling pathway can improve AMPKalfa phosphorylation, promoting oligodendrocyte myelination.
Connelly, et al. (80)	Folate (B9)	Clinical study	57 patients with probable Alzheimer’s disease (AD) were treated with cholinesterase inhibitors (ChI) and either folic acid of placebo concurrently.	The double blind study suggests that response to ChI in patients with Alzheimer’s disease may be improved by the use of folic acid.
Watanabe, et al. (81)	Folate (B9)	Clinical study	The study used the Japanese version of the Center for Epidemiological Studies Depression (CES-D) scale with 41 woman to screen for depressive symptoms and investigate the impact of dietary folate intake on these symptoms.	The study showed that depression was less incident in women whose dietary intake of folate exceeded 240 µg per day (the recommended dietary allowance), which indicated that folate supplementation could reduce the incidence of depression.
Zuhayr, et al. (82)	Cobalamin (B12)	Clinical study	The study gathered patients suffering from neurological disorders due to cobalamin deficiency with serum levels <200pg/mL. These patients were treated with 5mg of Hydroxocobalamin (Hdrx) three times per day for 10 days and later 3 doses per month. Serum levels were measured at days 0, 10 and 90.	The study observed that serum cobalamin increased dramatically from day 0 to 90. Some cases showed improvement of gait handicap, motor deficit, ataxia, depressive mood, impotence, and parenthesis, with no adverse effects notified during he treatment period.
Li, et al. (83)	Thiamine (B1) and cobalamin (B12)	*In vitro* and *in vivo*	*In vivo* studies utilized rats to establish a cerebral palsy model using hypoxia-ischemia induction. *In vitro* studies used mouse N2A neuroblastoma cells.	The supplementation of thiamine and cobalamin contributed to the mitigation of neuron apoptosis and nerve injury in cerebral palsy models.

As previously discussed, thiamine is crucial for cellular biochemistry, impacting energy metabolism, neurotransmission, and membrane function. Although thiamine deficiency is rare in developed countries, it can lead to severe neurological disorders, emphasizing the importance of adequate thiamine intake for optimal health and as treatment when administered promptly (6, 42).

## 3 Vitamin B2

Vitamin B2, also known as riboflavin, is predominantly found in milk and dairy products, as well as in organ meats such as liver and kidneys (5, 11, 43). Additionally, it is present in fatty fish and dark green vegetables (5, 10, 11). The recommended daily allowance of riboflavin varies by gender: 1.3 mg/day for males and 1.1 mg/day for females (5, 43). Riboflavin is absorbed in the small intestine, stomach, duodenum, colon, and rectum, through a carrier-mediated transport using riboflavin transporter 3, which has the main function of absorbing riboflavin acquired from dietary sources (43). Isolated riboflavin deficiency is rare and typically associated with risk factors including the acute or chronic consumption of alcohol, the use of birth control pills, and the administration of antimalarial agents and antidepressants, which can impair absorption (5, 44, 45).

Riboflavin plays a critical role in the biochemistry of the nervous system, supporting energy metabolism, since its depletion leads to the impairment of mitochondrial function, while also supporting antioxidant defense mechanisms, being essential for cellular respiration and immune function (10, 11, 43). In the body, through flavokinase and FAD synthetase, riboflavin is converted to its active forms, flavin mononucleotide (FMN) first, and then flavin adenine dinucleotide (FAD), which are essential for the synthesis of various compounds, including niacin, folic acid, vitamin B6, and heme proteins (1, 11, 43). Additionally, they play a critical role in the metabolism of fats into glucose, which is fundamental for the function of aerobic cells to generate energy (11, 43). This is even more important since FMN and FAD are integral components of the electron transport chain (ETC), a vital part of cellular respiration. In the electron transport chain (ETC), FMN and FAD function as cofactors that shuttle electrons, acting especially in I and II complexes, which contain flavoproteins reductases and electron transferring flavoproteins, thereby facilitating the production of adenosine triphosphate (ATP), the primary energy source of cells, being particularly important in the nervous system, due to its high energy demands (3, 43, 45). Studies prove that neurodegenerative diseases are highly associated with anomalies within complex I (45).

Riboflavin has therapeutic potential for certain mitochondrial disorders, such as mutations in the X-linked apoptosis-inducing factor mitochondria-associated 1 (AIFM1) and mitochondrial complex I deficiency nuclear type 20 or mitochondrial complex II deficiency (9, 43). Some mutations can lead to various neurological conditions, including neonatal mitochondrial encephalopathy, sensorineural hearing loss, axonal neuropathy, and, less commonly, motor neuron disease (9).

Riboflavin also supports the antioxidant defense system by aiding the regeneration of other antioxidants, such as glutathione, from oxidized glutathione (GSSG), since riboflavin, in the form of FAD, is necessary for glutathione reductase to play its role in converting GSSG (10, 43). Its capacity to neutralize reactive oxygen species (ROS) helps protect nerve cells from oxidative stress, a key factor in aging and neuro-degenerative conditions (10, 43). Consequently, riboflavin may significantly enhance cell longevity by boosting the activity and concentration of antioxidant enzymes, such as superoxide dismutase, catalase, and glutathione peroxidase, that act on deactivating peroxides, which may create an oxidant environment (10, 43). This antioxidant activity is vital because excessive ROS can impair mitochondrial function, leading to oxidative stress and affecting the energy production of neurons and glial cells, potentially causing migraine headaches (9). Therefore, riboflavin deficiency can have numerous consequences due to its crucial role in various cellular processes, as previously mentioned.

**Figure 4:**
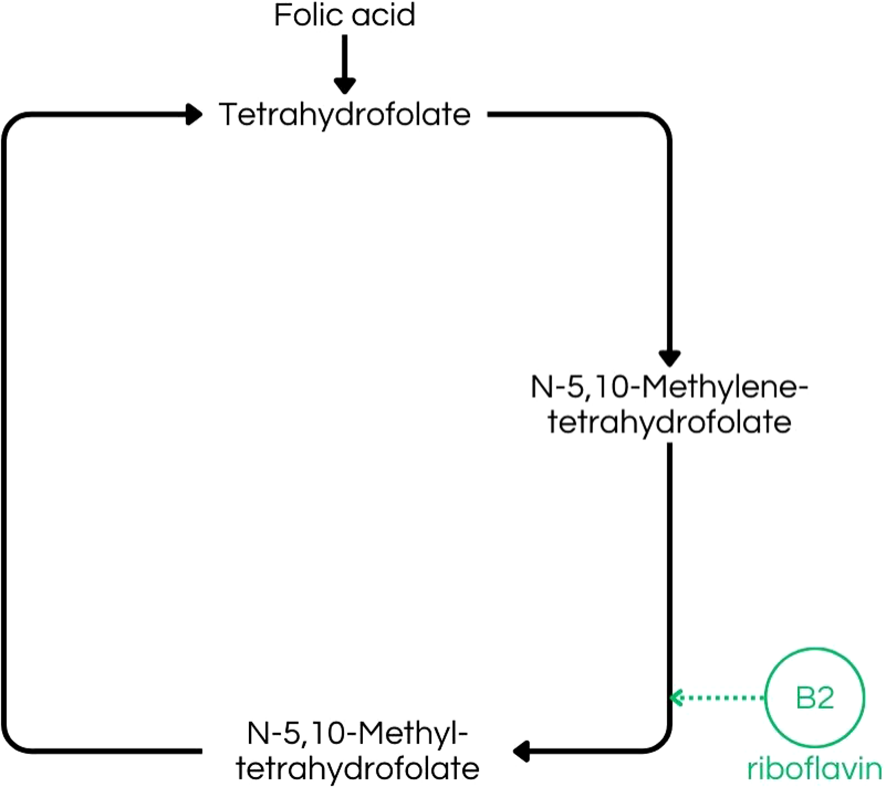
Folate-dependent remethylation process, highlighting riboflavin´s (B2) importance in the production of N-5,10-Methyltetrahydrofolate, a crucial step to S-adenosylmethionine (SAMe) production (43).

Vitamin B2 deficiency disrupts intermediary metabolism, potentially impairing energy production via the ETC, which is mainly concerned for the nervous system, given its high energy demands, and also can lead to disorders, such as migraine, because of energy failure, alongside overproduction of ROS (9, 47). Additionally, in different species, insufficient riboflavin intake can lead to peripheral demyelination (9, 47, 48). Riboflavin also plays a role in thyroxine metabolism, and its deficiency may contribute to mental illness, because riboflavin activates vitamin B6, leading to lower thyroxine levels, reduced hypothalamic serotonin synthesis, and decreased levels of S-adenosylmethionine (SAMe), because of disrupting the folate-methylation pathways involved in SAMe synthesis, all of which are associated with antidepressant effects, consequently, low levels of these compounds may cause depression (**Figure 4**) (48, 49). Besides, given its role as an antioxidant in the human body, riboflavin may act as a protective agent against neurological disorders, such as Parkinson’s Disease (DP), which has oxidative stress as a contributing factor in DP development (47).

Hoane et al. have shown that riboflavin may be therapeutic for traumatic brain injury. The study was conducted with 41 male rats that received contusion injuries or sham procedures. It improved the acquisition of reference and working memory while also reducing behavioral impairments.

In summary, riboflavin (vitamin B2) is essential for the biochemistry of the nervous system, functioning as a cofactor in redox reactions, supporting ATP production, enhancing antioxidant defense, and promoting overall cellular health (10). Finally, riboflavin deficiency may be reversible through dietary supplementation and adequate ingestion of this vitamin, which may prevent neurological disorders, such as migraine, besides depression (11, 47, 48).

## 4 Vitamin B3

Vitamin B3, commonly known as niacin, is predominantly found in meats, cereals, dairy products, fish, and eggs, and the recommended daily intake for niacin ranges from 15 mg/day to 20 mg/day for humans (5, 11, 14). Niacin deficiency is generally rare but can occur in cases of alcoholism, prolonged use of certain drugs such as levodopa and ethionamide, and malabsorptive syndromes, such as Crohn’s disease, major gastrointestinal surgeries or jejunoileitis (5, 12).

Niacin is crucial for the biochemistry of the nervous system, playing a significant role in energy metabolism, antioxidant activities, and the synthesis of essential molecules for neuronal function, such as acting in dopamine synthesis (13–15). It is metabolized from tryptophan into its active forms, nicotinamide adenine dinucleotide (NAD) and nicotinamide adenine dinucleotide phosphate (NADP) (1, 3, 11). The first one is synthesized through three steps, starting with the conversion of nicotinic acid into NAD, utilizing nicotinic acid mononucleotide and nicotinic acid adenine dinucleotide, while the second one is achieved by the action of enzyme NAD+ kinase, which phosphorylates NAD (3, 13, 14). These coenzymes are integral to numerous redox reactions that are vital for energy metabolism and the maintenance of cellular integrity, since NADP is crucial to cells’ metabolism, acting in anabolic metabolism, oxidant defense, and cytochrome P450 metabolism; while NAD participates in catabolic metabolism, Krebs cycle and glycolysis (14, 15). Additionally, both NAD and NADP are essential for cholesterol synthesis and DNA repair (3, 11, 13).

Nicotinamide adenine dinucleotide (NAD) is a co-substrate for various enzymes, such as sirtuins, polymerases, and cyclic ADP-ribose synthesis (14). Sirtuins, for example, are a family of proteins that act in cell metabolism, inflammation, and oxidative stress (13, 14). Additionally, NAD is a substrate for ADP-ribosyltransferases, which catalyze ADP-ribose transfer reactions, converting NAD into nicotinamide and ADP-ribosylated products (13–15). These reactions play a critical role in several cellular processes, including cell cycle progression, DNA repair, apoptosis, and aging (13, 14). Moreover, NAD has a crucial role in glycolysis, the citric acid cycle, and oxidative phosphorylation, since it acts as a proton acceptor, generating NADH, its reduced form, which collaborates with mitochondrial proton gradient (14). These metabolic pathways are essential for the production of adenosine triphosphate (ATP), via ATP synthase, the primary energy source of cells (14). Consequently, NAD is vital for maintaining the stability and integrity of genetic material within nerve cells, essential for the proper functioning and survival of neurons (13, 14).

Niacin also contributes to the synthesis of neurotransmitters, such as serotonin, a derivative of tryptophan, which is pivotal for mood regulation and various neurological functions, such as sleep and appetite, besides impacting cognitive and executive functions (13, 50). Furthermore, nicotinamide adenine dinucleotide phosphate (NADPH), derived from niacin, is crucial in antioxidant defense mechanisms, by aiding in the regeneration of other antioxidants, such as glutathione (13). This function is particularly important for protecting nerve cells from oxidative stress and mitigating its damage as well as preventing neuronal death, which acts as a prevention for Huntington’s disease and, when acting in the stimulation of glutathione redox cycle, attenuates cortical cell injury, in ischemic and traumatic injuries (13). Nicotinamide, a form of niacin, accelerates the differentiation of embryonic stem cells into post-mitotic neurons, influencing neurogenesis, mainly in early developmental stages (13, 51). In addition to its pro-differentiating actions, nicotinamide promotes neuronal survival, especially in oxidative stress environments, through various pathways, such as maintenance of protein kinase B (akt)-dependent phosphorylation of forkhead transcription factor (FOXO3a); and inhibition of caspase-3-mediated degradation of FOXO3a (13). Furthermore, NAD levels in the brain are essential for maintaining central nervous system (CNS) vascular integrity, due to NAD maintaining an energy supply that plays crucial roles in cell survival, such as having a protective factor in cerebral ischemia, since its disorder leads to energy depletion (13, 52). Alterations in NAD metabolism are critically linked to neurodegenerative disorders, as the accumulation of nicotinamide mononucleotide exerts pro-degenerative effects on axons, whereas enhancing its metabolism may rescue axons from degeneration (13, 53).

Chronic niacin deficiency leads to pellagra, a condition now rare in developed countries due to its association with poor nutrition, resulting from low intake of niacin or tryptophan (5, 11, 12). Pellagra is characterized by the “three Ds”: dementia, diarrhea, and dermatitis, with dermatitis being the only one associated with sun exposure, suggesting its relation to failure in DNA repair (5, 11, 12, 15). Additionally, it can manifest as memory loss, depression, disorientation, headache, and fatigue, which highlights niacin’s critical role in the growth and maintenance of the central nervous system. Without treatment, pellagra can be fatal, which is an important factor to be considered, since its low prevalence in developed countries causes missed diagnosis and delayed treatments (5, 11, 12, 54). In patients with alcohol dependence and delirium unresponsive to high doses of benzodiazepines, alcoholic pellagra should be considered, since chronic alcoholism leads to a lack of tryptophan 2,3-dioxygenase, an enzyme linked to niacin synthesis. Inhibition of this enzyme can lead to pellagra, which, in these cases, may present with cerebellar signs, myoclonus, and ataxia (5, 12, 55). Although rare in developed countries, pellagra remains endemic in poorer regions, with risk factors including low socioeconomic status, alcoholism, anorexia nervosa, cancer, and malabsorptive disorders, such as Crohn’s disease, as well as the use of some specific medications such as immunosuppressive and anti-tuberculosis drugs (12, 54).

Wang et al. proved, through an *in vivo* study with model rats and an *in vitro* study using organotypic hippocampal slice cultures, that nicotinamide mononucleotide improved learning and memory in Alzheimer’s disease rats while also reducing the accumulation of ROS and cell death in organotypic hippocampal slice cultures. In another study, Giri et al, showed that niacin may reduce inflammation by targeting its receptor GPR09A.

In summary, niacin is essential to the biochemistry of the nervous system, contributing to energy metabolism, neurotransmitter synthesis, such as serotonin and dopamine, antioxidant activities, and DNA repair (3, 11, 13, 56). Maintaining adequate levels of niacin is crucial to support the overall health and proper functioning of nerve cells, thereby preventing neuropsychiatric manifestations such as memory loss, headache, and depression.

## 5 Vitamin B6

Vitamin B6, or pyridoxine, is abundant in various dietary sources, including meat, fish, eggs, nuts, dairy products, non-citrus fruits such as bananas, and starchy vegetables like potatoes (5, 11). Additionally, it is synthesized by intestinal microflora (5). The recommended daily allowance for adults, regardless of gender is 1.3 mg/day (1). Since it is found in a wide range of foods, vitamin B6 deficiency due to dietary insufficiency is rare (11, 57). Deficiency in vitamin B6 is commonly linked to malnutrition, alcoholism, malabsorption syndromes, or as an adverse effect of certain medications, such as cyclosporine, hydralazine, cycloserine, penicillamine, isoniazid, levodopa, phenelzine and chemotherapy treatments (5, 11, 16, 18).

Pyridoxine is crucial for the biochemistry of the nervous system, playing a significant role in amino acid biosynthesis and degradation (57, 58). In addition to this, it is involved in sugar and fatty acid metabolism (17, 57). Intracellularly, pyridoxine is converted to its active form, pyridoxal phosphate (PLP), which is the only form of the vitamin capable of functioning as a cofactor for enzymes (6, 16, 18, 58). PLP acts as a cofactor for numerous enzymes involved in amino acid metabolism, particularly those associated with neurotransmitter synthesis, like serotonin, dopamine, and gamma-aminobutyric acid (GABA) (6, 11, 16–18, 57–59). In addition, it is an important coenzyme in the metabolism of carbohydrates, lipids, and proteins (11, 16, 18, 59). Consequently, pyridoxine significantly influences the adrenergic, serotonergic, and glutamatergic systems (6). Serotonin is influential on the central nervous system, affecting sleep, appetite, and cognitive functions, in addition to mood improvement (17). Dopamine acts on the sympathetic nervous system regulating blood pressure and heart rate (17). GABA is an inhibitory neurotransmitter (6, 17) that controls neuron excitability (17). As a consequence, low levels of vitamin B6 lead to reduced neurotransmitter synthesis, which can be associated with depression and dysfunctions of the brain, like epilepsy (17, 18).

**Figure 5:**
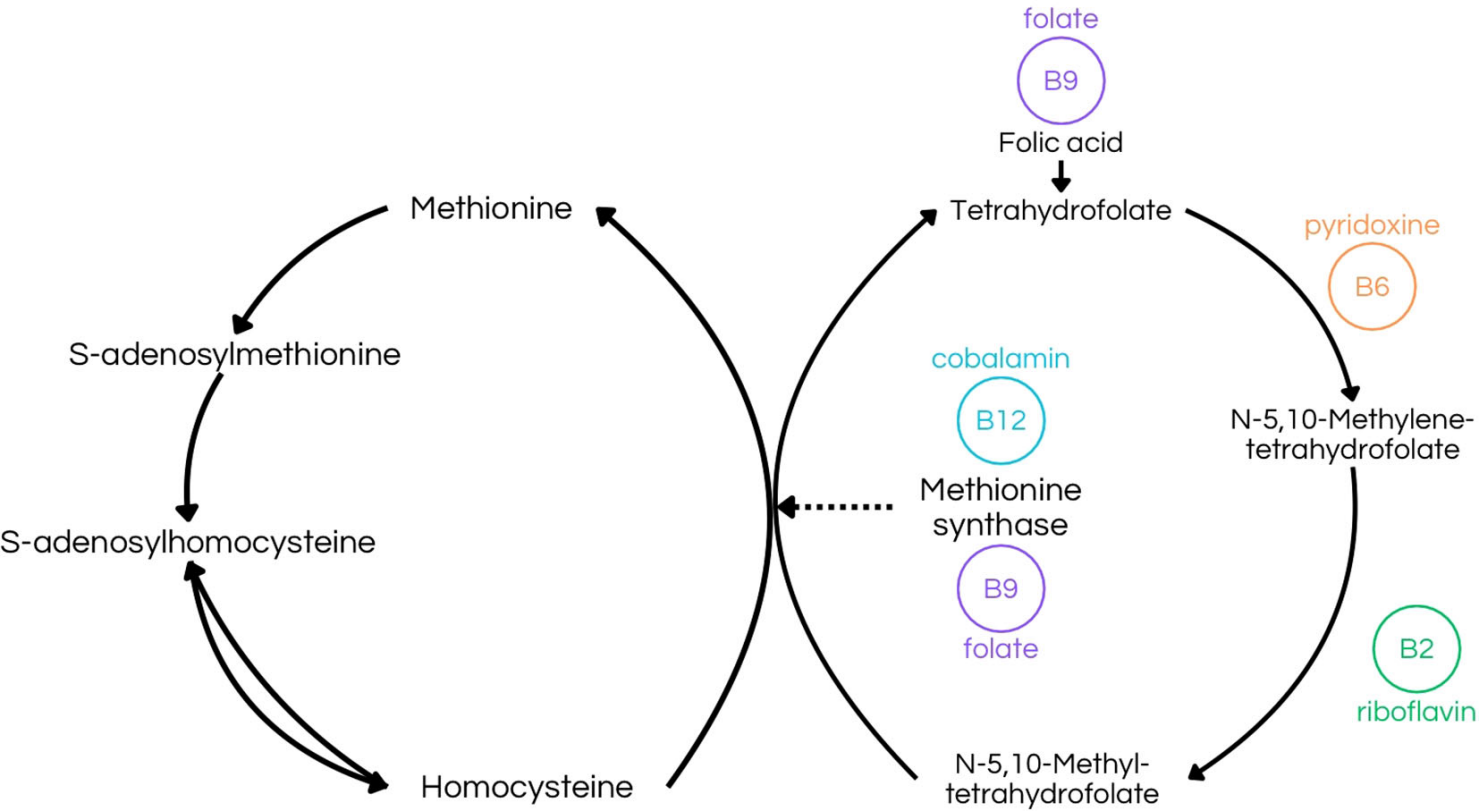
Transmethylation Pathway illustrating the folate-dependent remethylation process. This figure emphasizes the role of riboflavin (B2) in producing N-5,10-methyltetrahydrofolate, pyridoxine (B6) in synthesizing N-5,10-methylenetetrahydrofolate, and folate (B9) in forming tetrahydrofolate (8, 10, 26, 27, 32, 33). Additionally, it highlights the combined function of folate (B9) and cobalamin (B12) in the synthesis of methionine synthase (9, 13, 20, 22, 44, 61).

Pyridoxine may also exert a protective effect on the nervous system by regulating the glutamatergic system and thus modulating GABA and glutamate levels (6). Given that GABA is the primary inhibitory neurotransmitter, its deficiency can lead to severe consequences, such as seizures (4, 6). Conversely, elevated levels of glutamate, the precursor to GABA, can also result in similar neurological issues (6). Another crucial aspect of vitamin B6 is its essential role in gestation and postnatal brain development, likely due to its involvement in the regulation of GABA levels (6). Pyridoxine is also critical for heme synthesis, a key component of hemoglobin, and thus vitamin B6 is directly linked to an efficient oxygen transport to neuronal cells and overall brain function (60). Pyridoxal phosphate (PLP) is integral to one-carbon metabolism, a series of biochemical reactions necessary for the synthesis of nucleotides and other molecules essential for DNA and RNA production (6, 18, 57, 58). This involvement in nucleotide synthesis contributes to the maintenance of genetic material in nerve cells (6).Additionally, PLP plays a role in homocysteine metabolism, catalyzing the reaction that forms 5,10-methylene-THF, needed for the methylation of homocysteine to methionine,which also depends upon B2, B6, B9 and B12 vitamins (6, 11, 57, 58) (**Figure 5**). When deficient, it can increase the accumulation of homocysteine, which is significant in the development of cerebrovascular diseases and cognitive impairment (4, 20, 58).

Furthermore, pyridoxine is involved in myelin formation, serving as a cofactor in sphingolipid synthesis (6, 58, 59). Given these roles, vitamin B6 deficiency can severely impact both the central and peripheral nervous systems (6). Neurological symptoms range from impaired cognitive function, depression, seizures, and premature aging of neurons, to paresthesias, painful dysesthesias, and thermal sensations (6, 11, 16, 58). It has also been proposed that pyridoxine deficiency can have a role in the development of carpal tunnel syndrome, due to its critical role in peripheral nerve metabolism as a cofactor for neuronal protein synthesis (59). In infants, pyridoxine deficiency can cause irritability, abnormallyacute hearing, and seizures, which are unresponsive to anticonvulsants but respond to pyridoxine supplementation (5, 11).

A study performed by Yang and Wang in 2009 showed *in-vivo* study that pyridoxine can inhibit glutamate release from cerebrocortical synaptosomes by suppressing voltage-dependent Ca2+ entry. This result supports that vitamin B6 may function as a source for brain therapeutics against excitotoxicity, since the release of glutamate in excess can result in neuronal damage (78).

In conclusion, vitamin B6 plays a crucial role in the nervous system, serving as a cofactor in reactions related to amino acid metabolism and the synthesis of neurotransmitters such as dopamine, serotonin, and GABA, as well as playing a significant part in the methylation of homocysteine (6, 11, 16–18, 57, 58). As a result, pyridoxine deficiency can lead to relevant neuropsychiatric manifestations, such as reduced neurotransmitter synthesis, depression, epilepsy, premature aging of neurons, paresthesias, cerebrovascular diseases, cognitive impairment and carpal tunnel syndrome (4, 6, 11, 16–18, 20, 58) In contrast, pyridoxine may have therapeutic potential for treating depression or aggressive behavior, since pyridoxine plays a crucial role in the tryptophan-serotonin pathway (61, 62).

## 6 Vitamin B9

Vitamin B9, commonly referred to as folate or folic acid, is predominantly present in vegetables, particularly green leafy varieties, as well as fruits, nuts, beans, dairy products, liver, eggs, and seafood (4, 5, 63). The recommended daily allowance for folate is modest, at 400 µg per day (1, 22). Pure folate deficiency is relatively uncommon because of the influence of a wide array of factors on folate levels (5). However, certain populations are, at an increased risk, including individuals with alcoholism, pregnant women, and those adhering to restricted diets, such as those diagnosed with phenyl-ketonuria or small bowel disorders associated with malabsorption (5, 63).

Folate is essential for the proper functioning of the nervous system, playing a pivotal role in DNA synthesis, methylation reactions, and neurotransmitter production (4, 21, 22). Given its critical importance, vitamin B9 deficiency is associated with severe neurological complications, such as spinal cord and peripheral nerve disease, dementia, organic brain syndrome, and pyramidal tract damage (19, 21, 64). Comparatively, folate deficiency is notably prevalent in cases of depression, occurring twice as often as vitamin B12 deficiencies (20, 21, 30, 64). Studies have reported low serum folate levels in 15% to 38% of patients with depression and in at least 56% of patients with affective disorders (65). Symptoms related to folate deficiency include fatigue, apathy, insomnia, impaired concentration, and irritability (30).

The folate cycle is crucial for its role in the synthesis of S-adenosylmethionine (SAM) and the metabolism of vitamin B12 (20–22, 25, 30, 66). SAM serves as a major methyl group donor in numerous methylation reactions across various tissues, particularly within the central nervous system, working in reactions that form phospholipids and biogenic amines (21, 32). It was suggested by Reynolds and Stramentinoli that decreased SAM can result in less methylation of neuronal membrane phospholipids which can alter, for example, neurotransmitter function (19). Vitamin B9, for its role in SAM synthesis, is indirectly related to these methylation reactions and is necessary for its epigenetic function (20–22, 25). More importantly, the synthesis of SAM, which requires the folate cycle, is crucial in neuropsychiatry for its role as a therapeutic agent in the treatment of depression, and as an antidepressant in Parkinson’s disease (30, 65, 67).

The folate cycle also supplies carbon units for various metabolic pathways (21). For instance, 5,10-methylene-THF is utilized in the synthesis of purines and pyrimidines, which are essential for DNA and RNA synthesis and genetic function (21, 22, 66). In addition, folate is necessary in the synthesis of dTMP from dUMP (4, 22). When folate is deficient, dUMP starts to accumulate, which makes uracil incorporated into DNA instead of thymine (4, 22). This process results in DNA point mutation and breaks, in addition to micronucleus formation and chromosome breakage (4, 22, 66). The elevated DNA damage and altered DNA methylation are risks for cancer and play essential roles in neurological, and developmental abnormalities and cognitive defects (4, 22, 66). The current level considered sufficient for folate in plasma (i.e. 2,2 ng/mL or 4,8 nmol/L) and red blood cells (i.e. 132 ng/mL or 298 nmol/L), which are based only on the prevention of anemia, is significantly lower than what is necessary for the prevention of this DNA damage, that is minimized in i.e. 36 nmol/L for plasma and 938 nmol/L for red blood cells (22).

Folic acid is crucial during periods of rapid cell division and tissue growth, such as fetal development, and is therefore significant in enhancing childhood development outcomes, especially in the nervous system (68, 69). Folate deficiency during this period is associated with the development of autism spectrum disorders in children, neural tube irregularity, and brain development defects in the fetus and brain function in later life (4, 20, 30, 68, 69). In addition, treatment with folinic acid has shown results in autism spectrum individuals, by improving core behavioral deficits, and may minimize DNA uracil and homocysteine accumulation, which can avoid chromosome breaks, and, therefore, brain damage and cerebrovascular diseases (4, 20, 69).

Folate plays a role in transferring one-carbon units to various intermediates in DNA synthesis and methylation reactions, including the conversion of homocysteine to methionine (24). Folate deficiency leads to the accumulation of homocysteine which is associated with an increased risk of cerebrovascular diseases, depression, and dementia, with a focus on vascular dementia and Alzheimer’s disease (4, 20, 22). Folate is involved in the synthesis of neurotransmitters such as serotonin, dopamine, and norepinephrine, which are critical for mood regulation, cognitive function, and overall mental well-being (4, 23).

Literature indicates that nearly one-third of psychiatric and psychogeriatric hospital admissions exhibit low red cell folate levels or decreased serum folate levels, even in the absence of anemia or macrocytosis (20, 21). In these patients, vitamin B9 deficiency is primarily attributed to poor diet, malabsorption, chronic illness, or drugs such as alcohol and barbiturates (20, 21). Low serum folate in elderly patients has been found to result in impaired spatial copying skills, abstraction performance, and non-verbal abstract thinking, in comparison to age-matched individuals with normal serum folate concentration (4, 19). It has also been associated with psychogeriatric dis-ease, depression, high neurotoxin activity, and a decrease in intellectual function such as episodic and visuospatial memory, attention, and abstract reasoning (4, 20).Reynolds and colleagues found that 17 out of 24 hospitalized patients (71%) presenting severe folic acid deficiency were diagnosed with depression (46). Furthermore, early studies reviewed by Reynold and colleagues suggest that one-third of anemic patients would develop neuropsychiatric symptoms if folate deficiencies remained untreated (46). Many of these placebo-controlled studies have demonstrated that folic acid supplementation improved symptoms in severely depressed patients, elderly depressed and demented patients, and those with major acute depression or schizophrenia, particularly in individuals with RBC folate levels below 453 nmol/L (46). Reynolds and colleagues observed that patients with low serum folate levels undergoing treatment with various antidepressant drugs had poorer therapeutic outcomes (19, 30, 65).

In addition, Weng et al. found that the supplementation of folate during pregnancy and lactation can accelerate oligodendrocyte maturation, rescue oligodendrocyte death and differentiation defects, and promote oligodendrocyte myelination (79). Connelly et al. demonstrated how the use of folic acid can also improve patients’ response to other medications, such as cholinesterase inhibitors (ChI) for treating Alzheimer’s Disease (80). Finally, it was concluded by Watanabe et al. that folate supplementation can also reduce the incidence of depression, especially in women (81).

In summary, vitamin B9 is integral to the biochemistry of the nervous system, supporting DNA synthesis, methylation reactions, homocysteine regulation, myelin synthesis, and neuronal regeneration (21, 24, 32). As a result, folate deficiency can lead to many neuropsychiatric manifestations, such as depression, alterations in neurotransmitter function, DNA damage, as well as being associated with the development of autism spectrum disorders in children, neural tube irregularity and brain development defects (4, 19, 20, 22, 30, 66, 68, 69). Thus, adequate folate intake is essential for preventing depression, due to its role in the synthesis of dopamine and serotonin, as well as dementia, through the prevention of neuronal damage (23). Therefore, folate is crucial for the maintenance of nervous system health.

## 7 Vitamin B12

Vitamin B12, also known as cobalamin, plays a crucial role in the biochemistry of the nervous system, contributing to essential processes such as the synthesis of DNA, S-adenosylmethionine (SAM), and succinyl-CoA, all of which support nerve cell function and overall neurological health (20, 22, 27, 70, 71). This well-established importance underlies the role of vitamin B12 deficiency in mood imbalances, psychotic disorders, and neuronal health, particularly in elderly patients, where this deficiency is estimated to affect up to 40% of the population (25, 72). Cobalamin can be obtained from red meat, shellfish, poultry, and other animal-sourced foods, as well as from dairy products such as milk and eggs (27, 73).

The definition of vitamin B12 deficiency varies with the assay used but generally includes serum cobalamin levels below 148 pmol/L plus symptoms, or elevated serum homocysteine, or increased methylmalonic acid (MMA) (74). The daily intake of cobalamin is recommended to be 2.4 µg/day (1). Such deficiencies are caused by several factors, including pernicious anemia, which constitutes a minority of cases, poor dietary intake, or food-bound cobalamin malabsorption (FBCM) (63, 74). FBCM is currently considered to be the most common cause of vitamin B12 deficiency, caused by impairment in the release of the vitamin from food, commonly because of conditions such as gastritis, achlorhydria, gastrectomy, use of antacids, or proton pump inhibitors (PPIs), gastric atrophy in the elderly, and Helicobacter pylori infection (63, 74). Another known cause of cobalamin deficiency is Diphyllobothrium latum (D. latum), a tape-worm parasite of the intestinal tract that can take up free vitamin B12 faster than the body’s intrinsic factor that oversees B12 absorption (75).

At the cellular level, vitamin B12 is involved in DNA synthesis and neuroprotection, acting as a cofactor for enzymes critical to these processes, such as methionine synthase and methylmalonyl-CoA mutase (20, 27). One of the active forms of cobalamin, methylcobalamin, serves as a cofactor for methionine synthase, facilitating the conversion of homocysteine to methionine (7, 20, 25, 72, 73). Cobalamin deficiency results in the decrease of this conversion, resulting in high plasma levels of homocysteine, which is associated with a higher risk of cerebrovascular disease, depression, dementia, cellular stress, and apoptosis (25, 27, 64, 70). Methionine is an essential amino acid and a precursor for S-adenosylmethionine (SAM), a vital methyl donor in various biochemical reactions (19, 20, 25, 71, 76). Dietary methionine intake alone is insufficient to meet the total requirement for SAM synthesis, necessitating additional synthesis catalyzed by vitamin B12 (67). This methylation is crucial to regulate gene expression, neurotransmitter synthesis, myelin formation, and the methylation of histones and DNA (25, 70, 77). Recent studies have also shown that SAM can act as a therapeutic agent for treating depression, as an antidepressant in Parkinson’s disease, and in endogenous and bipolar patients with depression and hypomanic symptoms (19, 67). SAM is additionally required for phosphatidylcholine synthesis, which is abundant in neuronal membranes and is crucial for synaptogenesis and neurite outgrowth (25).

Vitamin B12 is a cofactor for the enzyme methylmalonyl-CoA mutase, which converts methylmalonyl-CoA to succinyl-CoA, a crucial step in fatty acid metabolism (25, 27, 73). Deficiency in vitamin B12 can lead to the accumulation of methylmalonyl-CoA, which is subsequently converted to methylmalonic acid (MMA) (27, 70, 73). Elevated MMA levels in patients with vitamin B12 deficiency can contribute to myelin damage (myelopathy) and, consequently, peripheral and autonomic neuropathy (27).

Cobalamin deficiency can lead to neurological manifestations such as progressive axonal demyelination, impaired peripheral and sensory nerve function, areflexia, loss of proprioception and vibration sensitivity, and subacute combined spinal cord degeneration (27, 70). Additionally, vitamin B12 deficiency can cause neurocognitive manifestations including depression, episodes of psychosis, delirium, dementia, poor memory performance, and cognitive impairment (20, 26, 27). Because of its role as an antioxidant, vitamin B12 deficiency can also result in lipids, proteins, and nucleic acids oxidation, which may cause the development of age-related diseases, such as Parkinson’s disease, Alzheimer’s disease, and type 2 diabetes (25).

In 1978, Carney and Sheffield found that 31% of patients suffering from depression had low serum vitamin B12 levels (65). Studies reviewed by Hector and Burton involving 91 patients with hypovitaminosis B12 who exhibited neuropsychiatric symptoms found that clinical improvement was achieved in many individuals through vitamin B12 replacement therapy (26). This suggests that numerous patients with mental health issues may suffer from psychiatric states due to vitamin B12 deficiency and could benefit from straightforward replacement therapy interventions (74). However, many remain undiagnosed, potentially leading to irreversible neurological consequences (74).

More recent studies, performed by Zouhayr et al., showed many neurological improvements after treatment with hydroxicobalamin (Hdrx) to patients suffering from neurological disorders due to vitamin B12 deficiency (82). Patients experienced advancement in the treatment of gait handicap, motor deficit and ataxia, as well as improvement in depressive moods, impotence and parenthesis (82). In addition, Li et al. prove the contribution of vitamin B1 and cobalamin supplementation to the mitigation of neuron apoptosis and nerve injury in cerebral palsy (83).

Finally, vitamin B12 is crucial for the biochemistry of the nervous system, participating in DNA synthesis, methionine synthesis, methylation reactions, myelin synthesis, and neurotransmitter production (20, 25, 27, 42, 70, 73, 77). Deficiencies in vitamin B12 can lead to neurological complications, including peripheral neuropathy, cognitive decline, and psychiatric diseases such as depression and psychosis (20, 26, 27). These points underscore the importance of maintaining adequate cobalamin levels.

## 8 Conclusion

Deficiencies in complex B vitamins, particularly vitamins B9 (folate) and B12 (cobalamin), have significant neuropsychiatric manifestations that can profoundly impact mental and neurological health. These deficiencies are implicated in a wide array of conditions, including depression, cognitive decline, psychosis, peripheral neuropathy, and myelopathy. The underlying mechanisms involve critical biochemical processes such as DNA synthesis, methylation reactions, neurotransmitter production, and myelin synthesis, all of which are essential for the proper functioning and maintenance of the nervous system. Given the high prevalence of B vitamin deficiencies in specific populations, such as the elderly and those with restrictive diets, there is a pressing need for heightened clinical awareness and early intervention. Ensuring adequate intake and timely diagnosis and treatment of B vitamin deficiencies can mitigate their neuropsychiatric effects and improve patient outcomes, highlighting the critical role of these vitamins in maintaining neurological health.
